# Supplementary material for: Facile Synthesis of Some Coumarin Derivatives and Their Cytotoxicity through VEGFR2 and Topoisomerase II Inhibition
Source: Molecules. 2022 Nov 28;27(23):8279. doi: 10.3390/molecules27238279 (PMC9737644; doi:10.3390/molecules27238279)
Supplement: Supplementary file 1 [file molecules-27-08279-s001.zip › molecules-2021190-supplementary.pdf]

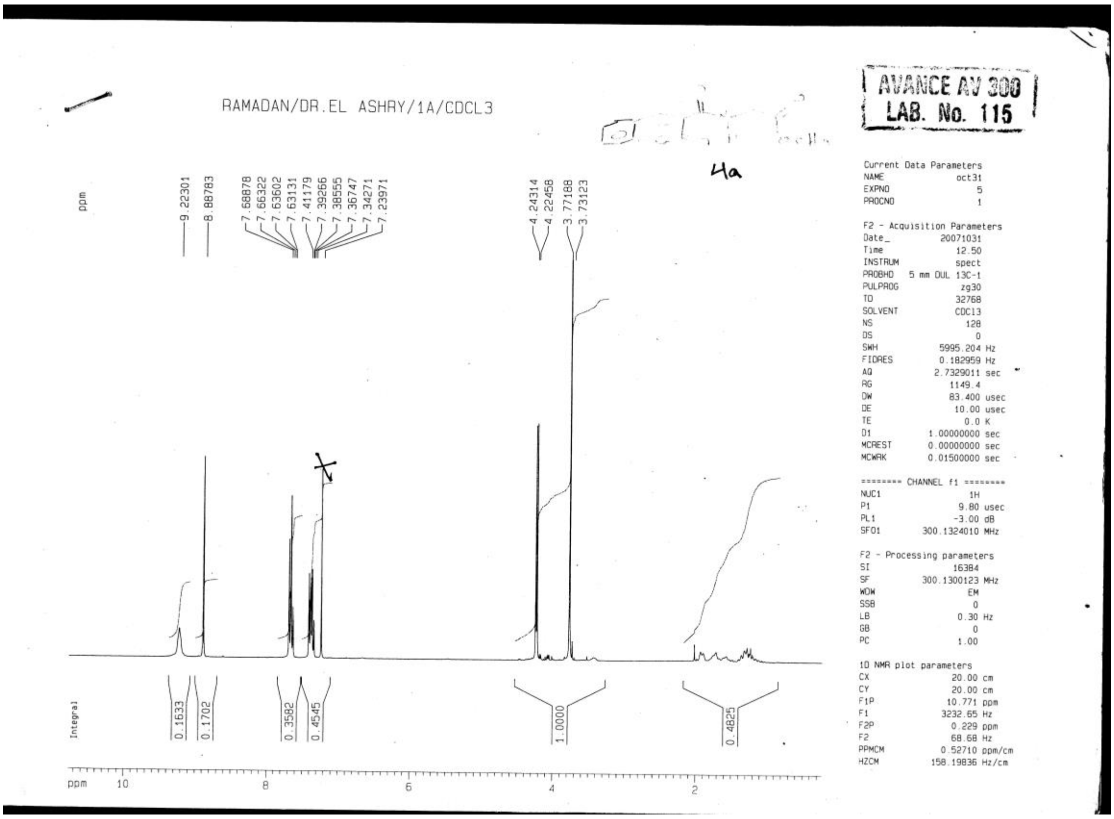

RAMADAN/DR.EL ASHRY/1A/CDCL3
4a
AVANCE AV 300
LAB. No. 115
9.22301
8.88783
7.68878
7.66322
7.63602
7.63131
7.41179
7.39266
7.38555
7.36747
7.34271
7.23971
4.24314
4.22458
3.77188
3.73123
Integral
0.1633
0.1702
0.3582
0.4545
1.0000
0.4825
ppm 10 8 6 4 2
Current Data Parameters
NAME oct31
EXPNO 5
PROCNO 1
F2 - Acquisition Parameters
Date_ 20071031
Time 12.50
INSTRUM spect
PROBHD 5 mm DUL 13C-1
PULPROG zg30
TD 32768
SOLVENT CDCl3
NS 128
DS 0
SWH 5995.204 Hz
FIDRES 0.182959 Hz
AQ 2.7329011 sec
RG 1149.4
DW 83.400 usec
DE 10.00 usec
TE 0.0 K
D1 1.00000000 sec
MCREST 0.00000000 sec
MCWRK 0.01500000 sec
======== CHANNEL f1 ========
NUC1 1H
P1 9.80 usec
PL1 -3.00 dB
SFO1 300.1324010 MHz
F2 - Processing parameters
SI 16384
SF 300.1300123 MHz
WDW EM
SSB 0
LB 0.30 Hz
GB 0
PC 1.00
1D NMR plot parameters
CX 20.00 cm
CY 20.00 cm
F1P 10.771 ppm
F1 3232.65 Hz
F2P 0.229 ppm
F2 68.68 Hz
PPMCM 0.52710 ppm/cm
HZCM 158.19836 Hz/cm

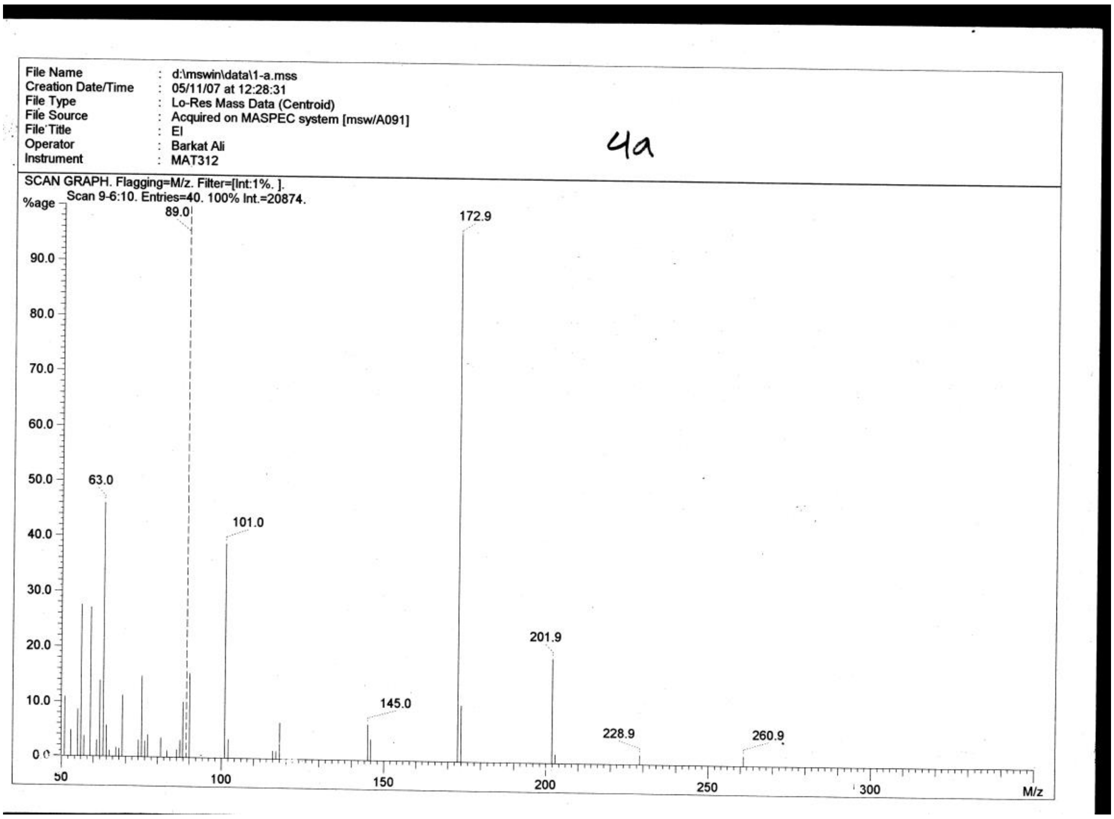

File Name : d:\mswin\data\1-a.mss
Creation Date/Time : 05/11/07 at 12:28:31
File Type : Lo-Res Mass Data (Centroid)
File Source : Acquired on MASPEC system [msw/A091]
File Title : EI
Operator : Barkat Ali
Instrument : MAT312
4a
SCAN GRAPH. Flagging=M/z. Filter=[Int:1%.].
Scan 9-6:10. Entries=40. 100% Int.=20874.
%age
90.0
80.0
70.0
60.0
50.0
40.0
30.0
20.0
10.0
0.0
89.0
172.9
63.0
101.0
201.9
145.0
228.9
260.9
50 100 150 200 250 300 M/z

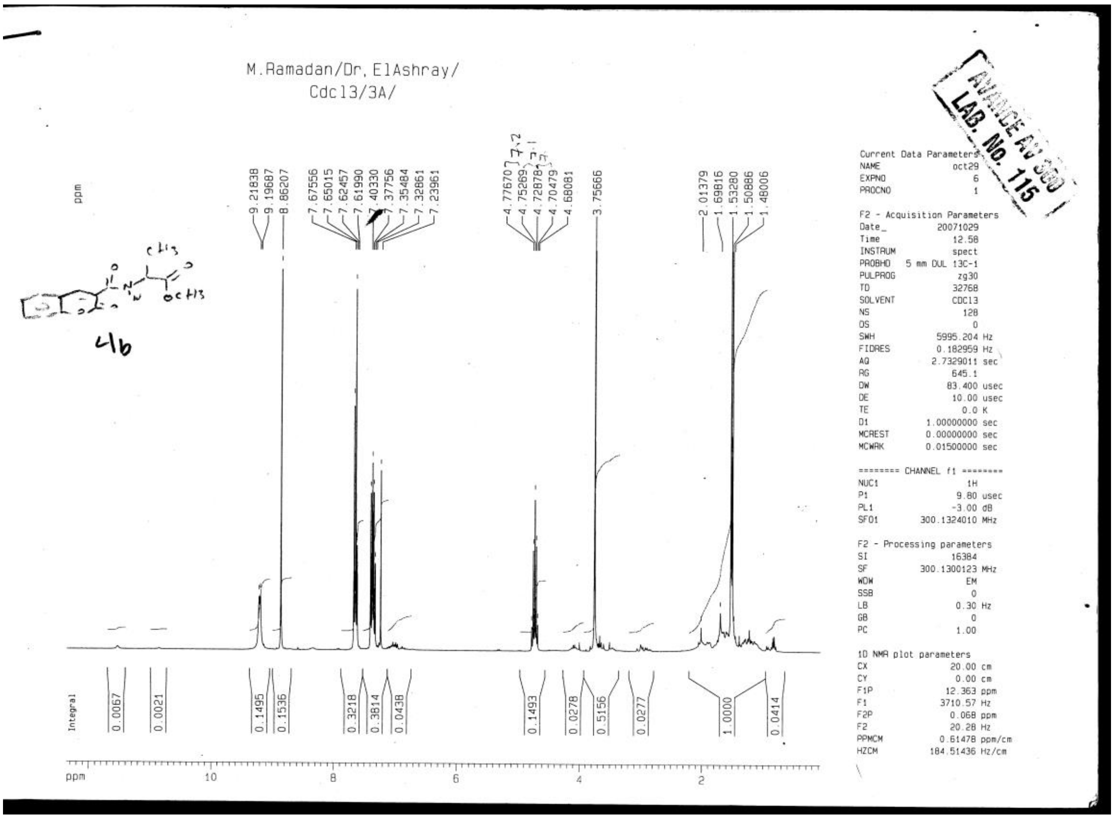
M.Ramadan/Dr,ElAshray/
Cdcl3/3A/
4b
9.21838
9.19687
8.86207
7.67556
7.65015
7.62457
7.61990
7.40330
7.37756
7.35484
7.32861
7.23961
4.77670
4.75269
4.72878
4.70479
4.68081
3.75666
2.01379
1.69816
1.53280
1.50886
1.48006
Integral
0.0067
0.0021
0.1495
0.1536
0.3218
0.3814
0.0438
0.1493
0.0278
0.5156
0.0277
1.0000
0.0414
ppm
10
8
6
4
2
AVANCE AV 300
LAB. No. 115
Current Data Parameters
NAME oct29
EXPNO 6
PROCNO 1
F2 - Acquisition Parameters
Date_ 20071029
Time 12.58
INSTRUM spect
PROBHD 5 mm DUL 13C-1
PULPROG zg30
TD 32768
SOLVENT CDC13
NS 128
DS 0
SWH 5995.204 Hz
FIDRES 0.182959 Hz
AQ 2.7329011 sec
RG 645.1
DW 83.400 usec
DE 10.00 usec
TE 0.0 K
D1 1.00000000 sec
MCREST 0.00000000 sec
MCWRK 0.01500000 sec
======== CHANNEL f1 ========
NUC1 1H
P1 9.80 usec
PL1 -3.00 dB
SFO1 300.1324010 MHz
F2 - Processing parameters
SI 16384
SF 300.1300123 MHz
WDW EM
SSB 0
LB 0.30 Hz
GB 0
PC 1.00
1D NMR plot parameters
CX 20.00 cm
CY 0.00 cm
F1P 12.363 ppm
F1 3710.57 Hz
F2P 0.068 ppm
F2 20.28 Hz
PPMCM 0.61478 ppm/cm
HZCM 184.51436 Hz/cm

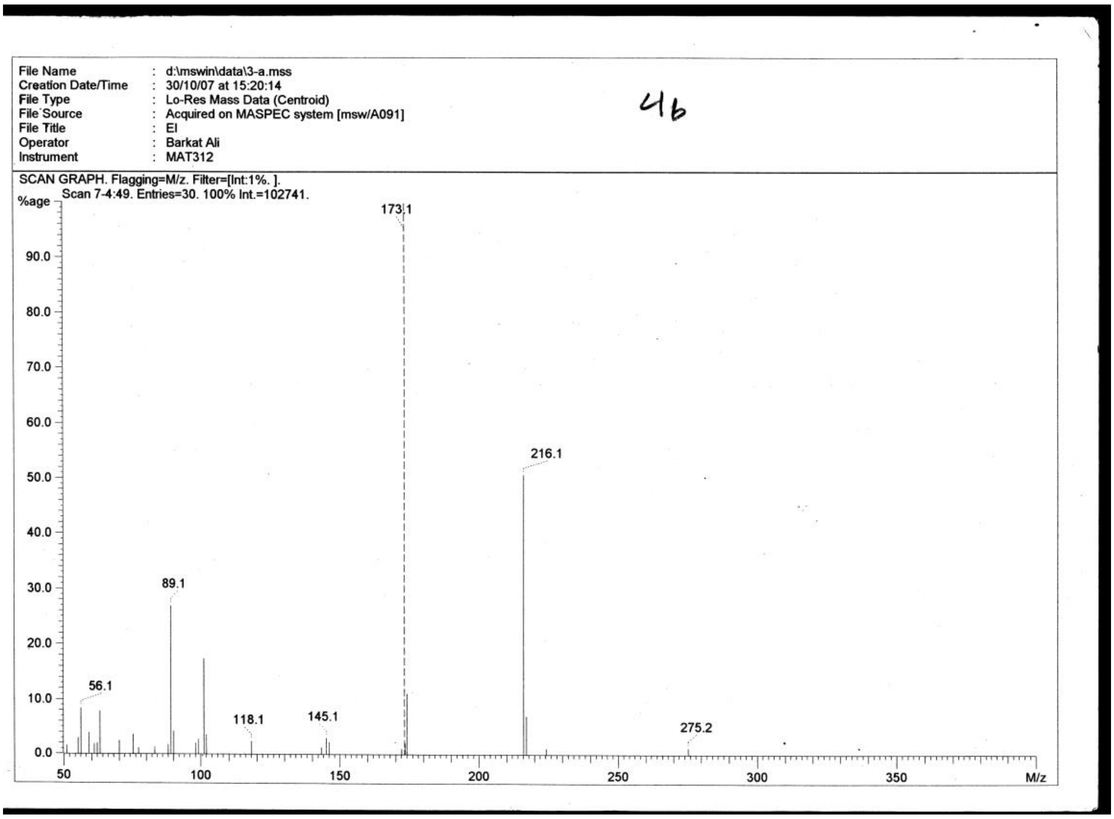
File Name : d:\mswin\data\3-a.mss
Creation Date/Time : 30/10/07 at 15:20:14
File Type : Lo-Res Mass Data (Centroid)
File Source : Acquired on MASPEC system [msw/A091]
File Title : EI
Operator : Barkat Ali
Instrument : MAT312
4b
SCAN GRAPH. Flagging=M/z. Filter=[Int:1%.].
Scan 7-4:49. Entries=30. 100% Int.=102741.
%age
173.1
216.1
89.1
56.1
118.1
145.1
275.2
M/z

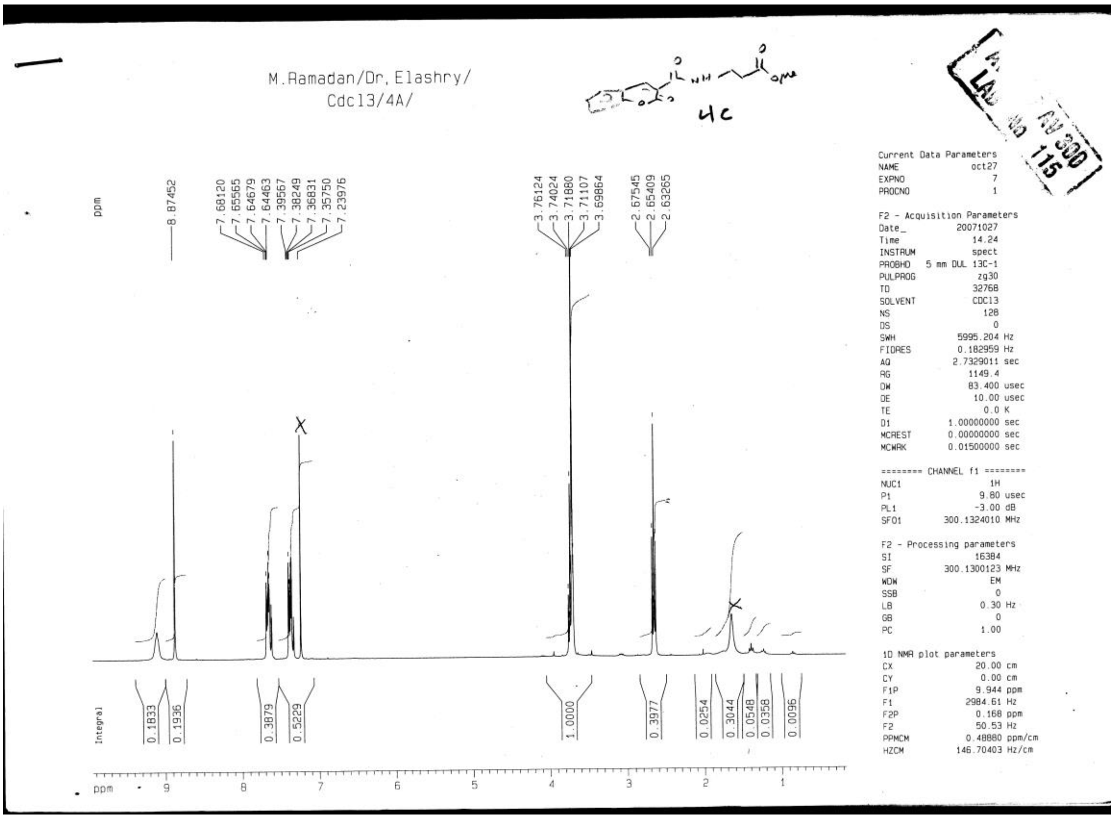
M.Ramadan/Dr,Elashry/
Cdcl3/4A/
4c
8.87452
7.68120
7.65565
7.64679
7.64463
7.39567
7.38249
7.36831
7.35750
7.23976
3.76124
3.74024
3.71880
3.71107
3.69864
2.67545
2.65409
2.63265
Integral
0.1833
0.1936
0.3879
0.5229
1.0000
0.3977
0.0254
0.3044
0.0548
0.0358
0.0096
ppm
9
8
7
6
5
4
3
2
1
Current Data Parameters
NAME oct27
EXPNO 7
PROCNO 1
F2 - Acquisition Parameters
Date_ 20071027
Time 14.24
INSTRUM spect
PROBHD 5 mm DUL 13C-1
PULPROG zg30
TD 32768
SOLVENT CDCl3
NS 128
DS 0
SWH 5995.204 Hz
FIDRES 0.182959 Hz
AQ 2.7329011 sec
RG 1149.4
DW 83.400 usec
DE 10.00 usec
TE 0.0 K
D1 1.00000000 sec
MCREST 0.00000000 sec
MCWRK 0.01500000 sec
======== CHANNEL f1 ========
NUC1 1H
P1 9.80 usec
PL1 -3.00 dB
SFO1 300.1324010 MHz
F2 - Processing parameters
SI 16384
SF 300.1300123 MHz
WDW EM
SSB 0
LB 0.30 Hz
GB 0
PC 1.00
1D NMR plot parameters
CX 20.00 cm
CY 0.00 cm
F1P 9.944 ppm
F1 2984.61 Hz
F2P 0.168 ppm
F2 50.53 Hz
PPMCM 0.48880 ppm/cm
HZCM 146.70403 Hz/cm
AV 300
115

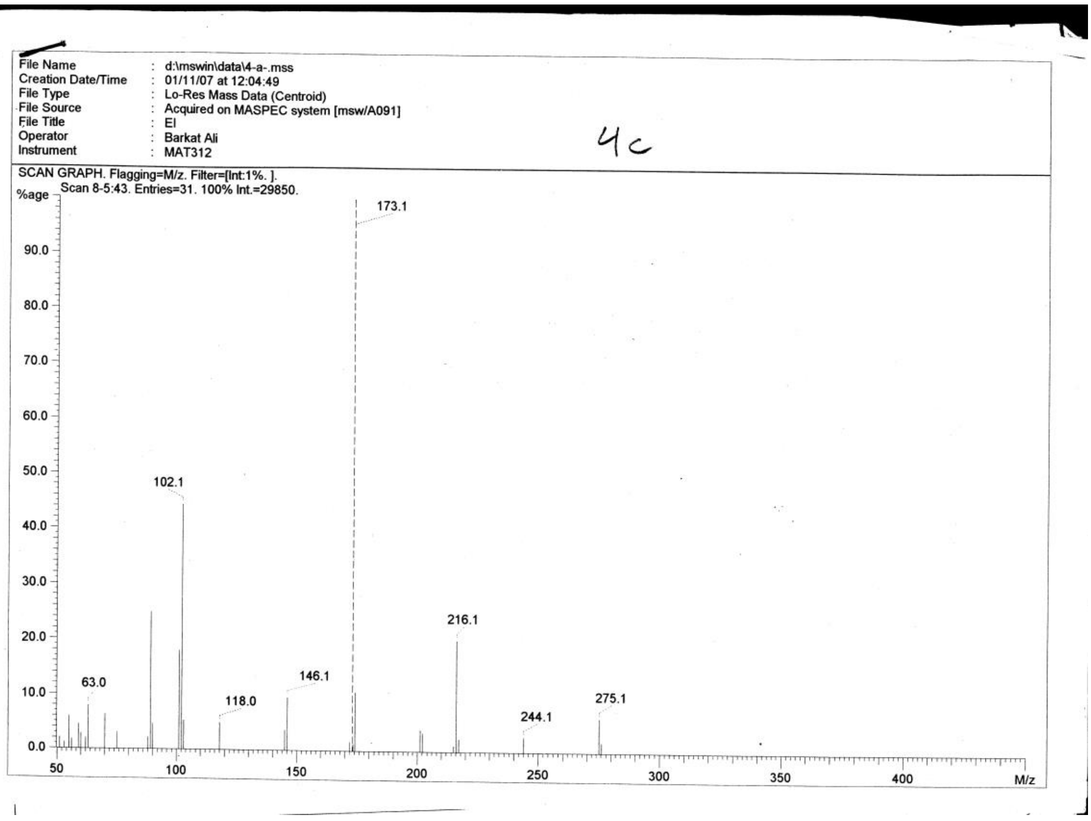
File Name : d:\mswin\data\4-a-.mss
Creation Date/Time : 01/11/07 at 12:04:49
File Type : Lo-Res Mass Data (Centroid)
File Source : Acquired on MASPEC system [msw/A091]
File Title : EI
Operator : Barkat Ali
Instrument : MAT312
4c
SCAN GRAPH. Flagging=M/z. Filter=[Int:1%.].
Scan 8-5:43. Entries=31. 100% Int.=29850.
%age
90.0
80.0
70.0
60.0
50.0
40.0
30.0
20.0
10.0
0.0
173.1
102.1
216.1
63.0
146.1
118.0
275.1
244.1
50
100
150
200
250
300
350
400
M/z

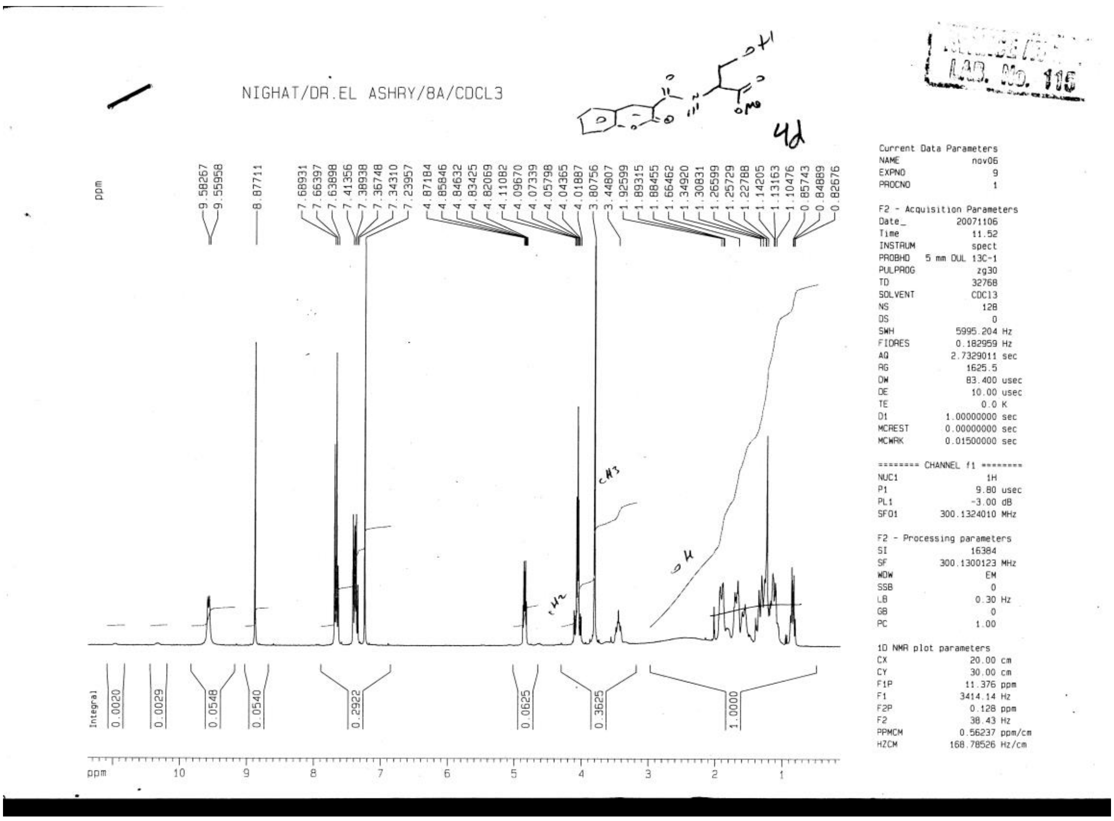
NIGHAT/DR.EL ASHRY/8A/CDCL3
4d
LAB. No. 115
9.58267
9.55958
8.87711
7.68931
7.66397
7.63898
7.41356
7.38938
7.36748
7.34310
7.23957
4.87184
4.85846
4.84632
4.83425
4.82069
4.11082
4.09670
4.07339
4.05798
4.04365
4.01887
3.80756
3.44807
1.92599
1.89315
1.88455
1.66462
1.34920
1.30831
1.26599
1.25729
1.22788
1.14205
1.13163
1.10476
0.85743
0.84889
0.82676
CH3
CH2
OH
Integral
0.0020
0.0029
0.0548
0.0540
0.2922
0.0625
0.3625
1.0000
ppm
10 9 8 7 6 5 4 3 2 1
Current Data Parameters
NAME nov06
EXPNO 9
PROCNO 1
F2 - Acquisition Parameters
Date_ 20071106
Time 11.52
INSTRUM spect
PROBHD 5 mm DUL 13C-1
PULPROG zg30
TD 32768
SOLVENT CDCl3
NS 128
DS 0
SWH 5995.204 Hz
FIDRES 0.182959 Hz
AQ 2.7329011 sec
RG 1625.5
DW 83.400 usec
DE 10.00 usec
TE 0.0 K
D1 1.00000000 sec
MCREST 0.00000000 sec
MCWRK 0.01500000 sec
======== CHANNEL f1 ========
NUC1 1H
P1 9.80 usec
PL1 -3.00 dB
SFO1 300.1324010 MHz
F2 - Processing parameters
SI 16384
SF 300.1300123 MHz
WDW EM
SSB 0
LB 0.30 Hz
GB 0
PC 1.00
1D NMR plot parameters
CX 20.00 cm
CY 30.00 cm
F1P 11.376 ppm
F1 3414.14 Hz
F2P 0.128 ppm
F2 38.43 Hz
PPMCM 0.56237 ppm/cm
HZCM 168.78526 Hz/cm

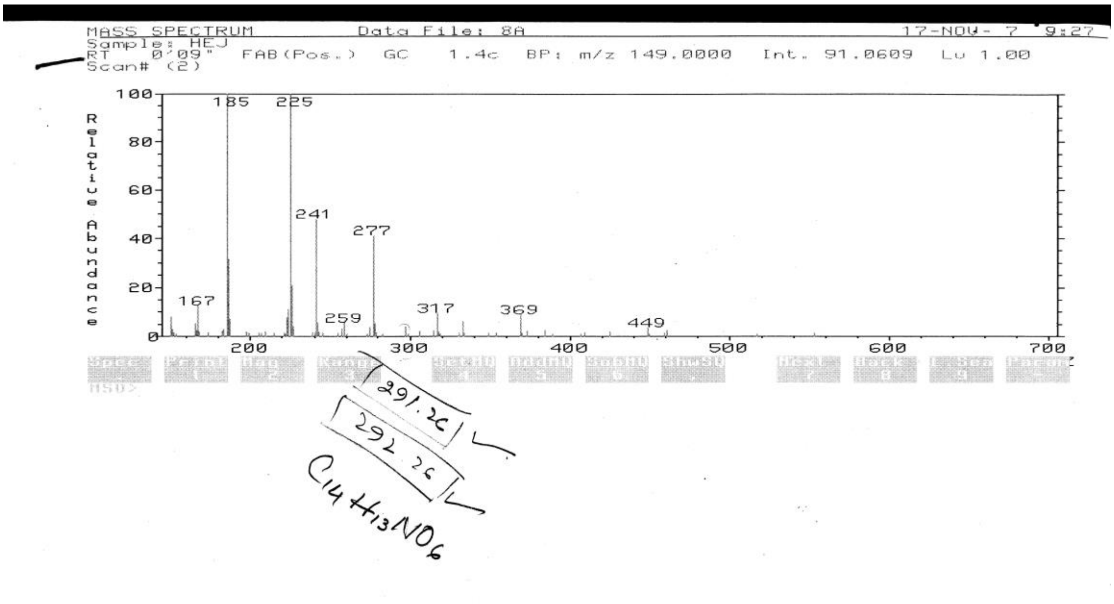
MASS SPECTRUM Data File: 8A 17-NOV- 7 9:27
Sample: HEJ
RT 0'09" FAB(Pos.) GC 1.4c BP: m/z 149.0000 Int. 91.0609 Lu 1.00
Scan# (2)
Relative Abundance
100
80
60
40
20
0
185
225
241
277
167
259
317
369
449
200 300 400 500 600 700
291.26
292.26
$C_{14}H_{13}NO_6$

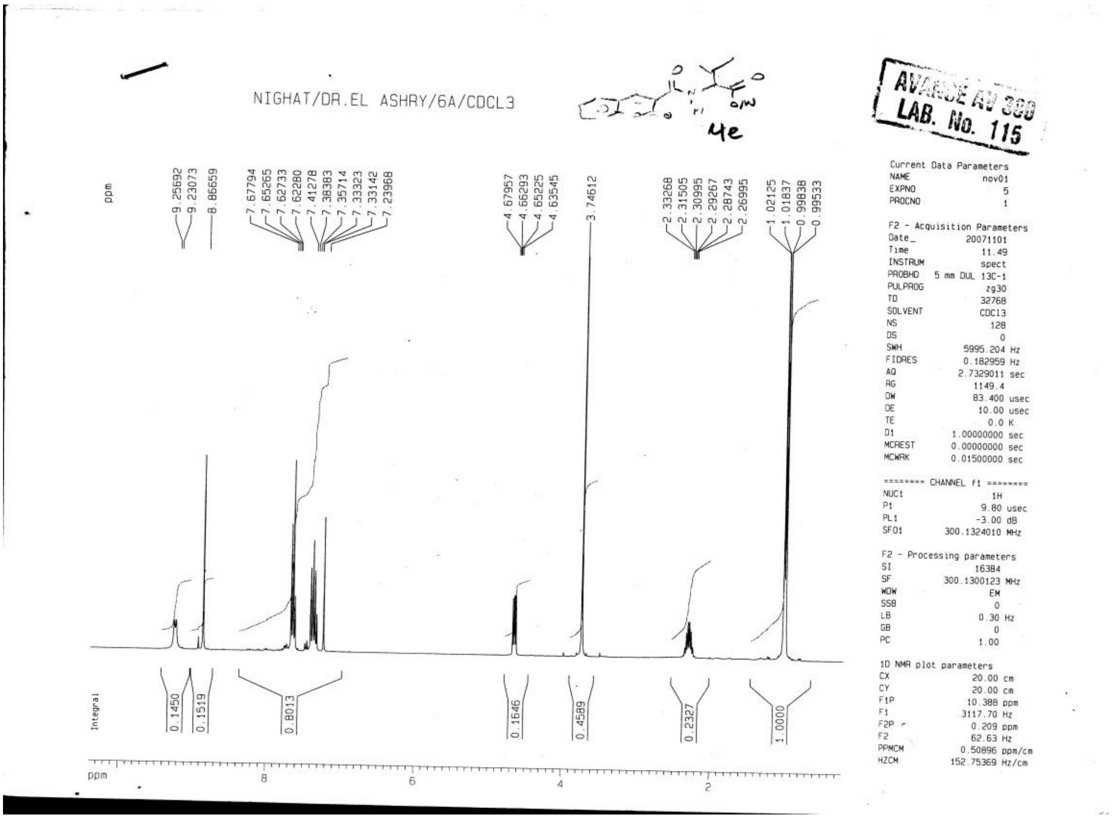
NIGHAT/DR.EL ASHRY/6A/CDCL3
4e
AVANCE AV 300
LAB. No. 115
ppm
9.25692
9.23073
8.86659
7.67794
7.65265
7.62733
7.62280
7.41278
7.38383
7.35714
7.33323
7.33142
7.23968
4.67957
4.66293
4.65225
4.63545
3.74612
2.33268
2.31505
2.30995
2.29267
2.28743
2.26995
1.02125
1.01837
0.99838
0.99533
Integral
0.1450
0.1519
0.8013
0.1646
0.4589
0.2327
1.0000
Current Data Parameters
NAME nov01
EXPNO 5
PROCNO 1
F2 - Acquisition Parameters
Date_ 20071101
Time 11.49
INSTRUM spect
PROBHD 5 mm DUL 13C-1
PULPROG zg30
TD 32768
SOLVENT CDCl3
NS 128
DS 0
SWH 5995.204 Hz
FIDRES 0.182959 Hz
AQ 2.7329011 sec
RG 1149.4
DW 83.400 usec
DE 10.00 usec
TE 0.0 K
D1 1.00000000 sec
MCREST 0.00000000 sec
MCWRK 0.01500000 sec
======== CHANNEL f1 ========
NUC1 1H
P1 9.80 usec
PL1 -3.00 dB
SFO1 300.1324010 MHz
F2 - Processing parameters
SI 16384
SF 300.1300123 MHz
WDW EM
SSB 0
LB 0.30 Hz
GB 0
PC 1.00
1D NMR plot parameters
CX 20.00 cm
CY 20.00 cm
F1P 10.388 ppm
F1 3117.70 Hz
F2P 0.209 ppm
F2 62.63 Hz
PPMCM 0.50896 ppm/cm
HZCM 152.75369 Hz/cm
ppm
8
6
4
2

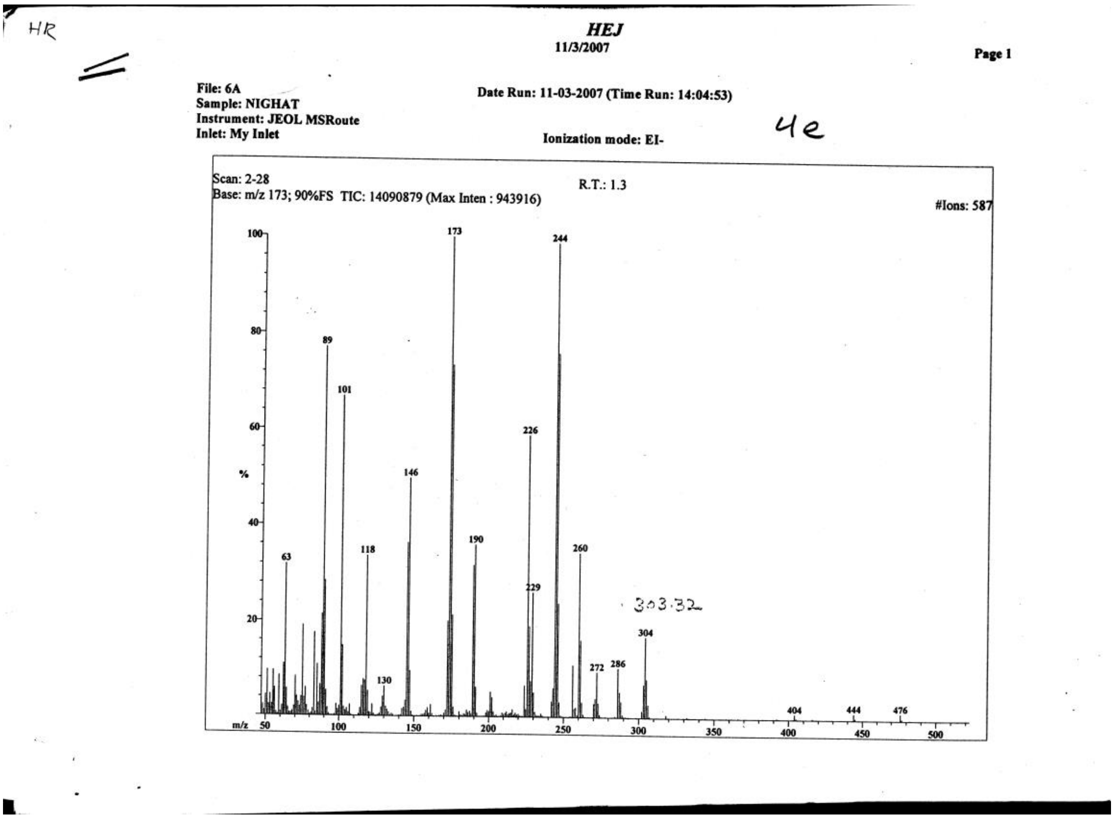
HR
HEJ
11/3/2007
Page 1
File: 6A
Sample: NIGHAT
Instrument: JEOL MSRoute
Inlet: My Inlet
Date Run: 11-03-2007 (Time Run: 14:04:53)
Ionization mode: EI-
4e
Scan: 2-28
Base: m/z 173; 90%FS TIC: 14090879 (Max Inten : 943916)
R.T.: 1.3
#Ions: 587
100
80
60
%
40
20
63
89
101
118
130
146
173
190
226
229
244
260
272
286
304
303.32
404
444
476
m/z
50
100
150
200
250
300
350
400
450
500

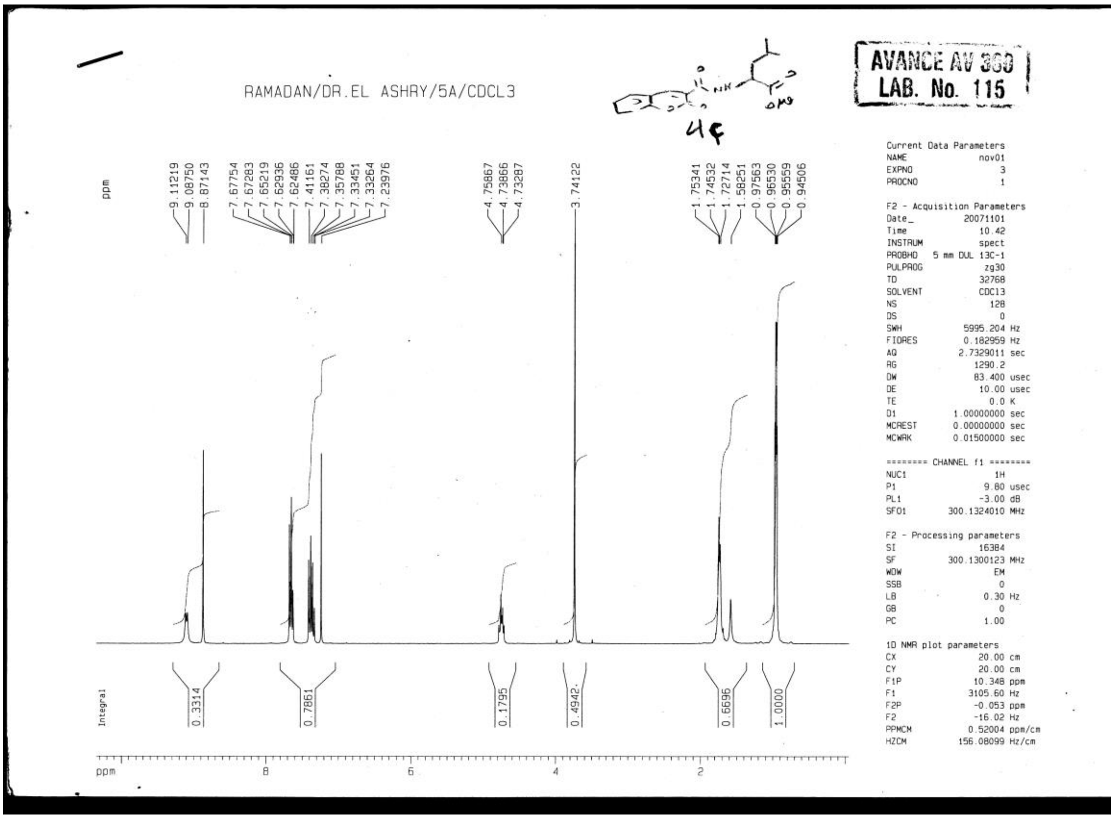

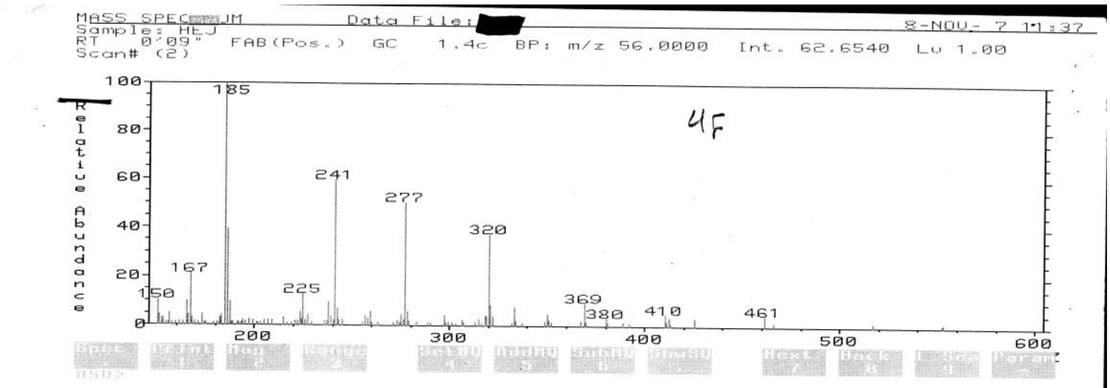

Ramadan

Mwt = 317.34

?

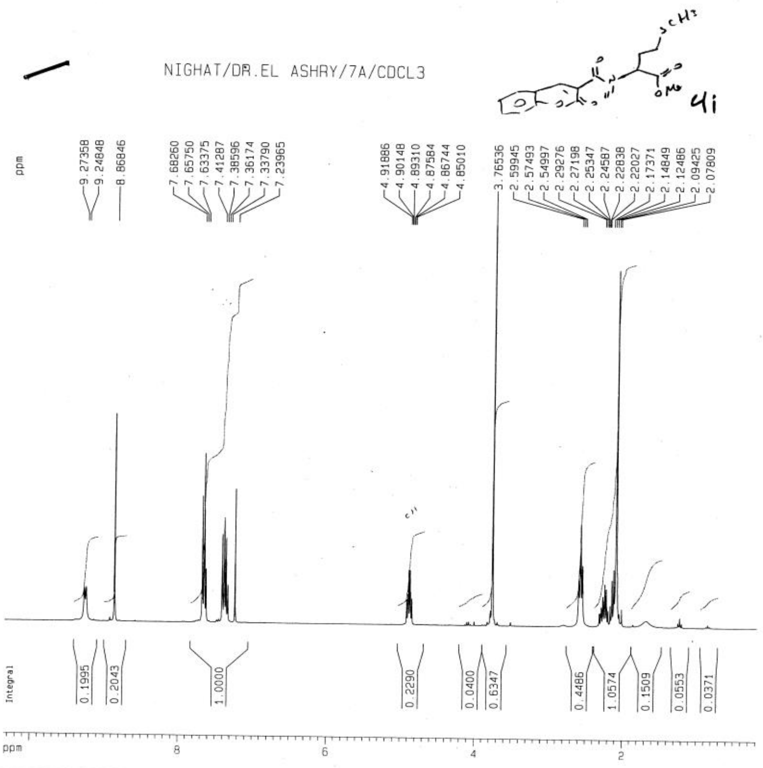

AVANCE AV 300
LAB. No. 115

Current Data Parameters
NAME nov01
EXPNO 7
PROCNO 1

F2 - Acquisition Parameters
Date_ 20071101
Time 12.55
INSTRUM spect
PROBHD 5 mm DUL 13C-1
PULPROG zg30
TD 32768
SOLVENT CDCl3
NS 128
DS 0
SWH 5995.204 Hz
FIDRES 0.182959 Hz
AQ 2.7329011 sec
RG 1149.4
DW 83.400 usec
DE 10.00 usec
TE 0.0 K
D1 1.00000000 sec
MCREST 0.00000000 sec
MCWRK 0.01500000 sec

======== CHANNEL f1 ========
NUC1 1H
P1 9.80 usec
PL1 -3.00 dB
SFO1 300.1324010 MHz

F2 - Processing parameters
SI 16384
SF 300.1300123 MHz
WDW EM
SSB 0
LB 0.30 Hz
GB 0
PC 1.00

1D NMR plot parameters
CX 20.00 cm
CY 20.00 cm
F1P 10.348 ppm
F1 3105.60 Hz
F2P 0.189 ppm
F2 56.58 Hz
PPMCM 0.50795 ppm/cm
HZCM 152.45122 Hz/cm

HEJ
11/1/2007

File: 7A
Sample: NIGHAT
Instrument: JEOL MSRoute
Inlet: My Inlet

Date Run: 11-01-2007 (Time Run: 12:42:17)

4i

Ionization mode: EI+

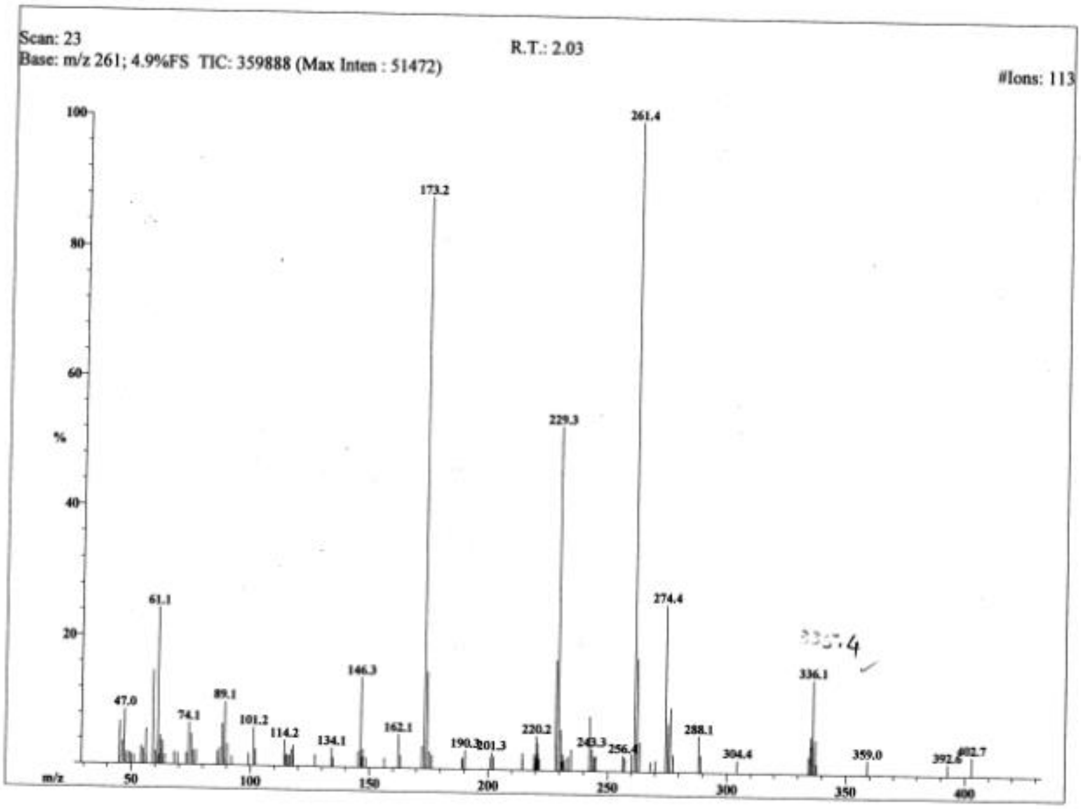

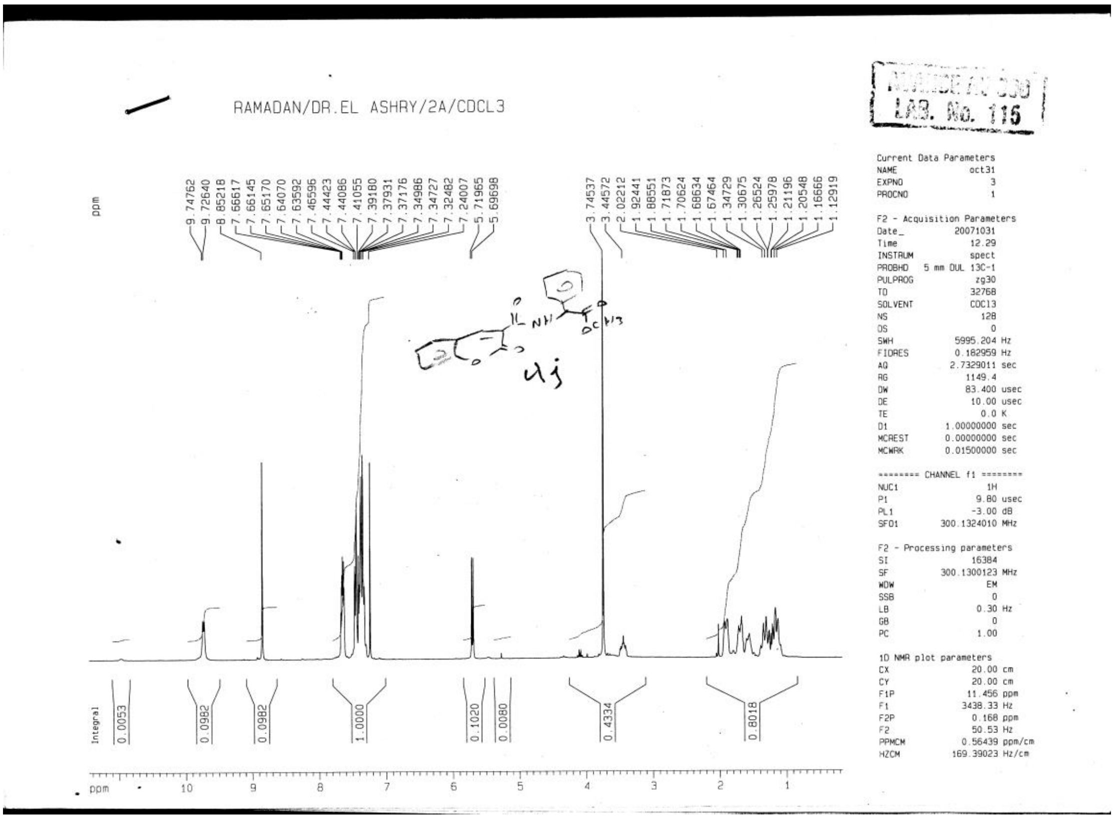
RAMADAN/DR.EL ASHRY/2A/CDCL3
LAB. No. 115
4j
9.74762
9.72640
8.85218
7.66617
7.66145
7.65170
7.64070
7.63592
7.46596
7.44423
7.44086
7.41055
7.39180
7.37931
7.37176
7.34986
7.34727
7.32482
7.24007
5.71965
5.69698
3.74537
3.44572
2.02212
1.92441
1.88551
1.71873
1.70624
1.68634
1.67464
1.34729
1.30675
1.26524
1.25978
1.21196
1.20548
1.16666
1.12919
Integral
0.0053
0.0982
0.0982
1.0000
0.1020
0.0080
0.4334
0.8018
ppm
Current Data Parameters
NAME oct31
EXPNO 3
PROCNO 1
F2 - Acquisition Parameters
Date_ 20071031
Time 12.29
INSTRUM spect
PROBHD 5 mm QUL 13C-1
PULPROG zg30
TD 32768
SOLVENT CDCl3
NS 128
DS 0
SWH 5995.204 Hz
FIDRES 0.182959 Hz
AQ 2.7329011 sec
RG 1149.4
DW 83.400 usec
DE 10.00 usec
TE 0.0 K
D1 1.00000000 sec
MCREST 0.00000000 sec
MCWRK 0.01500000 sec
======== CHANNEL f1 ========
NUC1 1H
P1 9.80 usec
PL1 -3.00 dB
SFO1 300.1324010 MHz
F2 - Processing parameters
SI 16384
SF 300.1300123 MHz
WDW EM
SSB 0
LB 0.30 Hz
GB 0
PC 1.00
1D NMR plot parameters
CX 20.00 cm
CY 20.00 cm
F1P 11.456 ppm
F1 3438.33 Hz
F2P 0.168 ppm
F2 50.53 Hz
PPMCM 0.56439 ppm/cm
HZCM 169.39023 Hz/cm

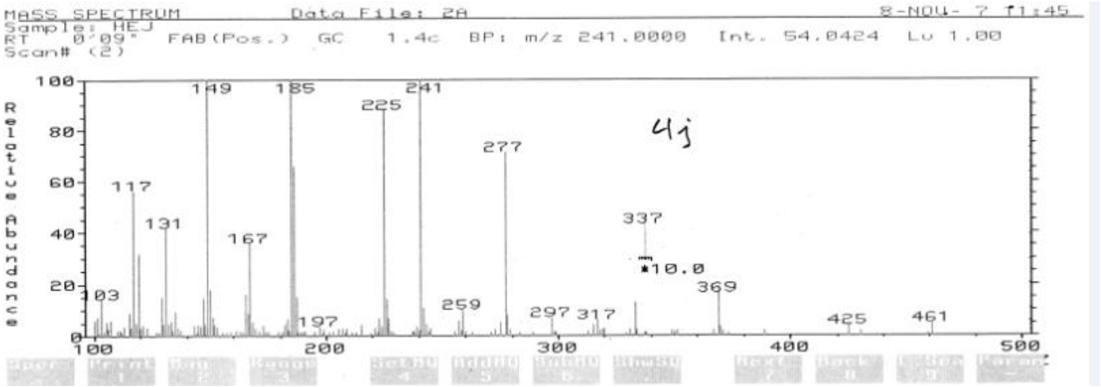
MASS SPECTRUM Data File: 2A 8-NOV- 7 11:45
Sample: HEJ
RT 0'09" FAB(Pos.) GC 1.4c BP: m/z 241.0000 Int. 54.0424 Lv 1.00
Scan# (2)
Relative Abundance
4j
103
117
131
149
167
185
197
225
241
259
277
297
317
337
*10.0
369
425
461

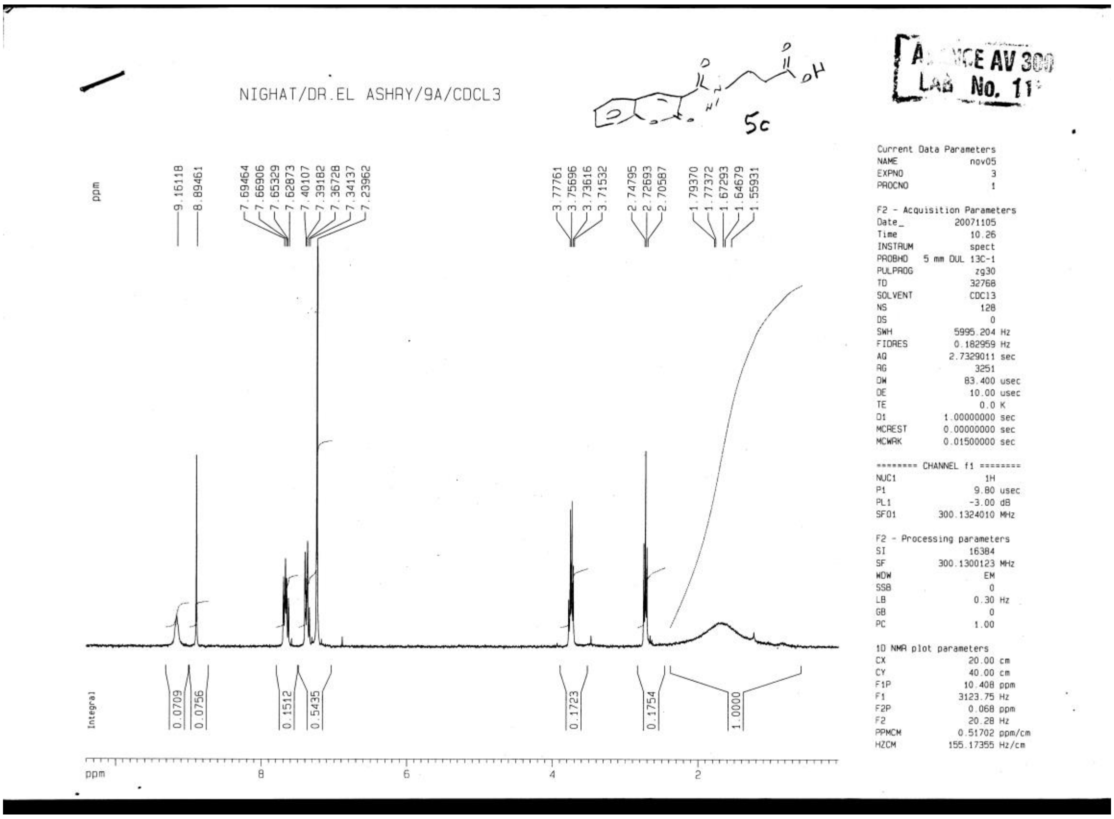

HEJ

11/1/2007

Page 1

File: 9A
Sample: NIGHAT
Instrument: JEOL MSRoute
Inlet: My Inlet

Date Run: 11-01-2007 (Time Run: 14:19:05)

Ionization mode: EI+

5c

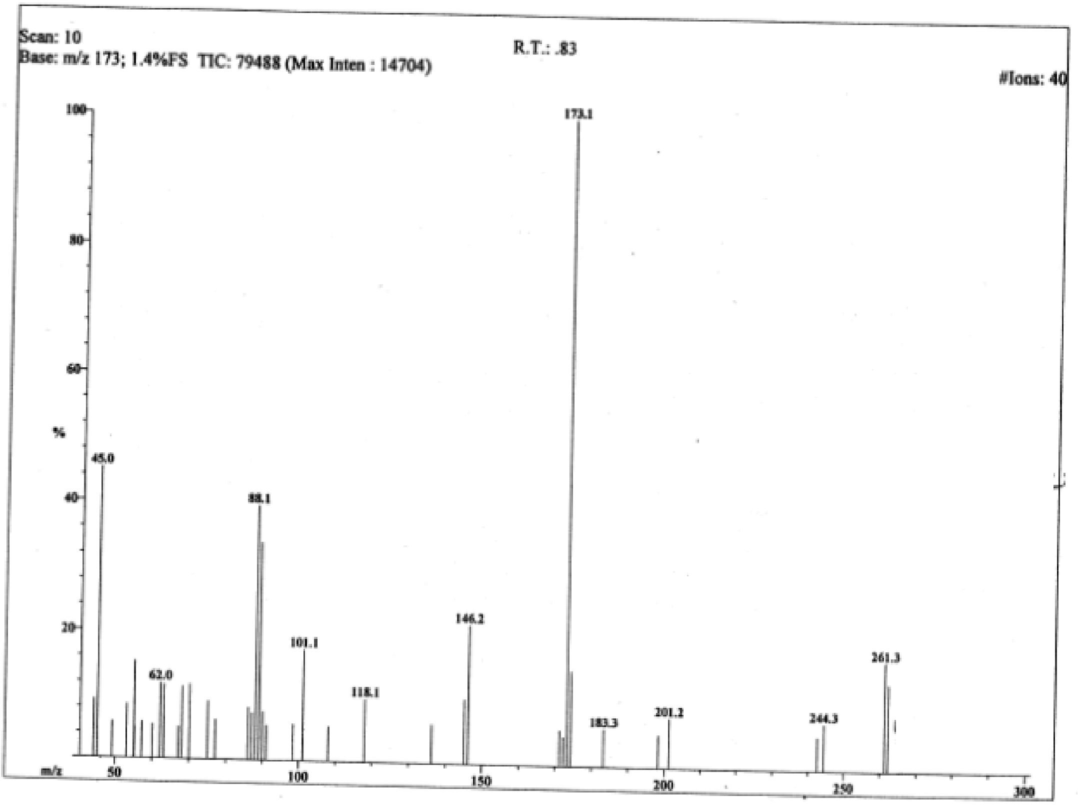

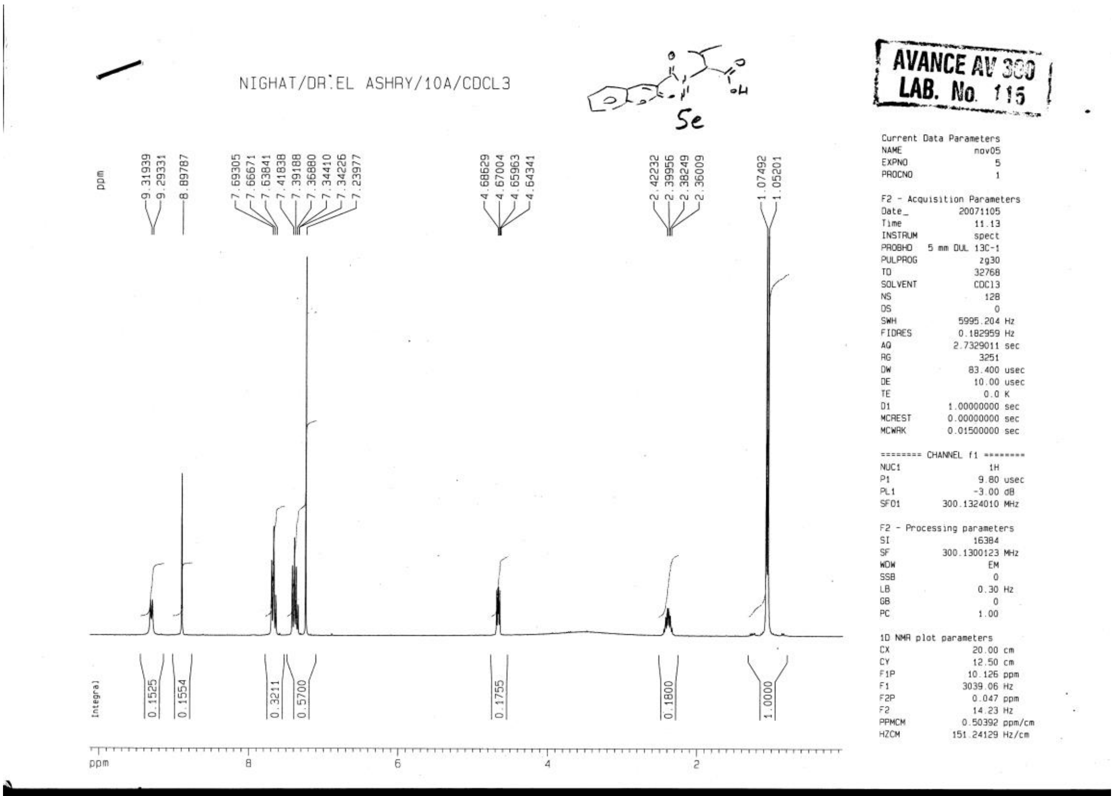

HEJ
11/1/2007

Pag

File: 10A
Sample: NIGHAT
Instrument: JEOL MSRoute
Inlet: My Inlet

Date Run: 11-01-2007 (Time Run: 14:08:57)

Ionization mode: EI+

5e

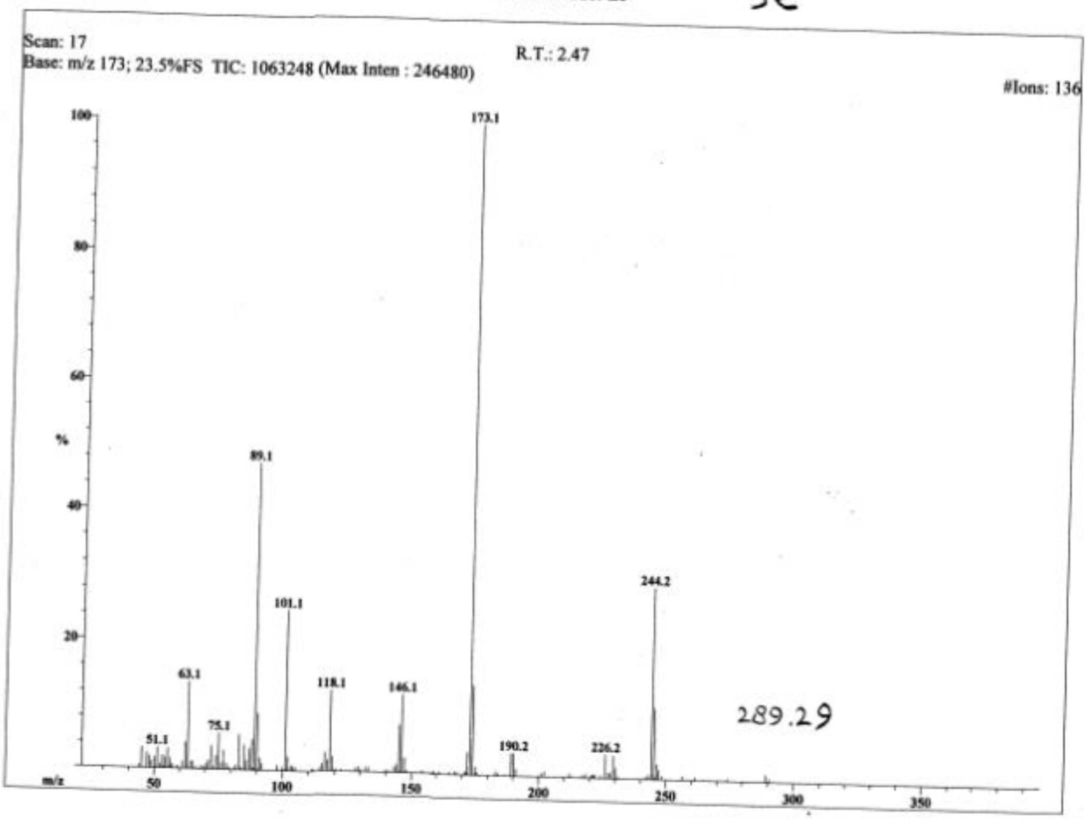

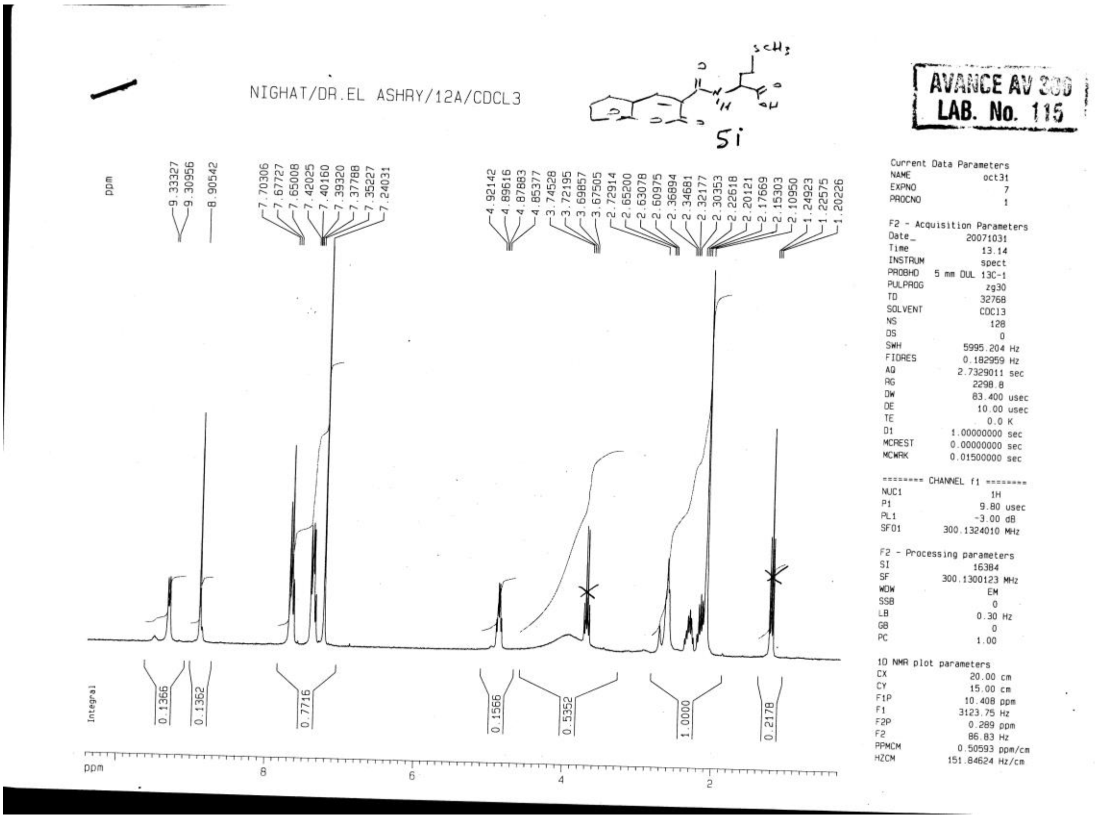

HEJ
11/1/2007

Page 1

File: 12A
Sample: NIGHAT
Instrument: JEOL MSRoute
Inlet: My Inlet

Date Run: 11-01-2007 (Time Run: 13:59:50)

Ionization mode: EI+ 5i

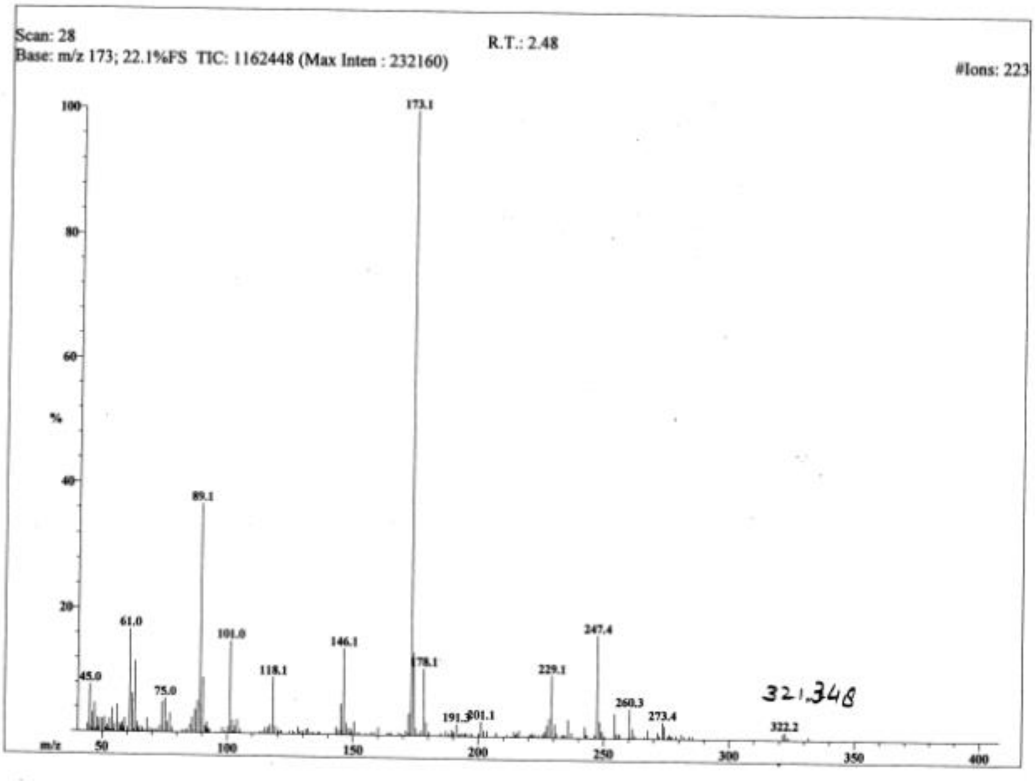

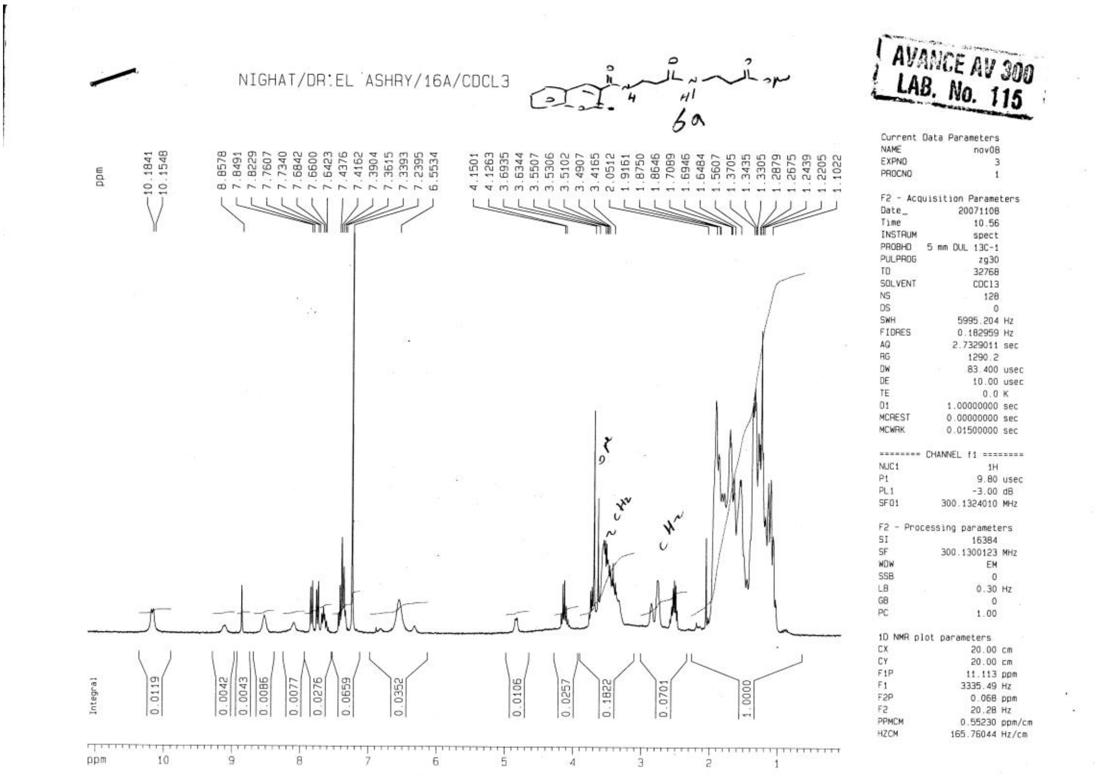

HEJ

11/3/2007

Page 1

File: 16A
Sample: NIGHAT
Instrument: JEOL MSRoute
Inlet: My Inlet

Date Run: 11-03-2007 (Time Run: 14:40:31)

Ionization mode: EI-

6a

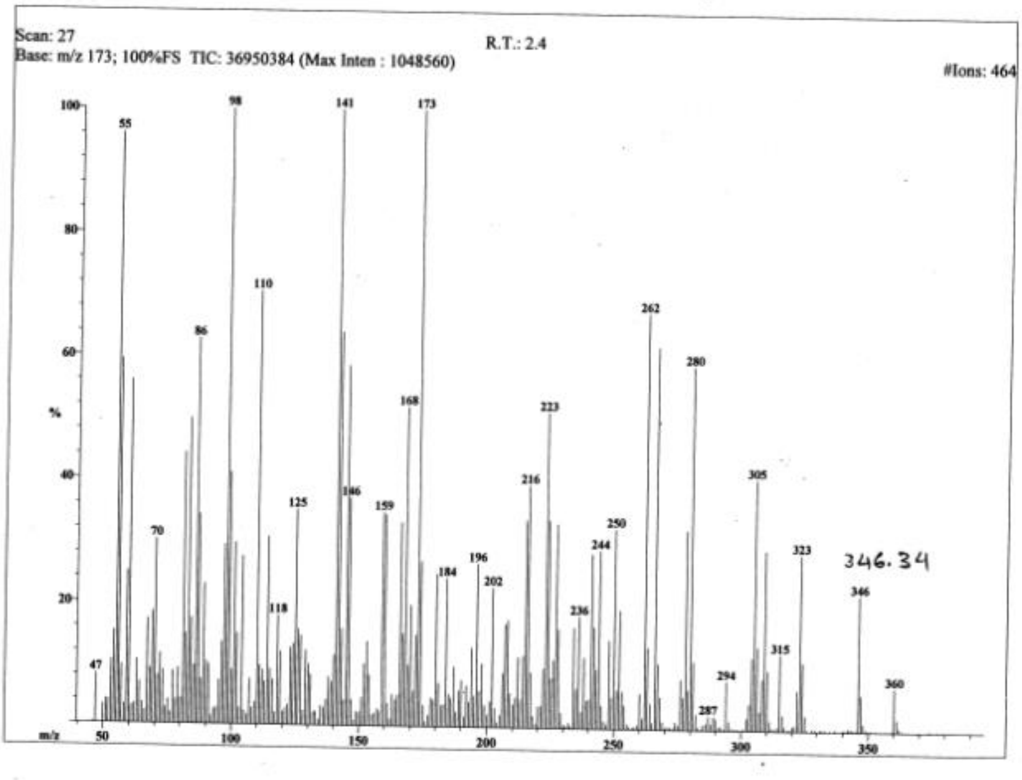

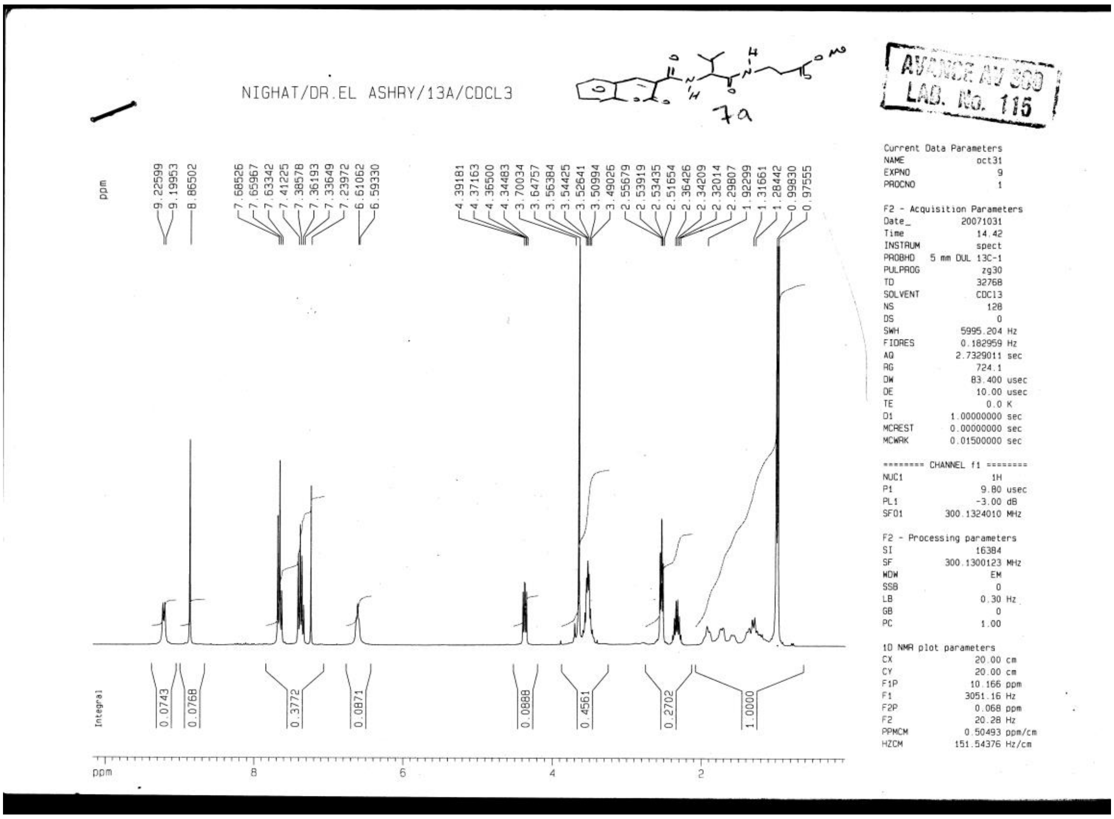

HEJ
11/3/2007

Page 1

File: 13A
Sample: NIGHAT
Instrument: JEOL MSRoute
Inlet: My Inlet

Date Run: 11-03-2007 (Time Run: 14:12:08)

Ionization mode: EI-

7a

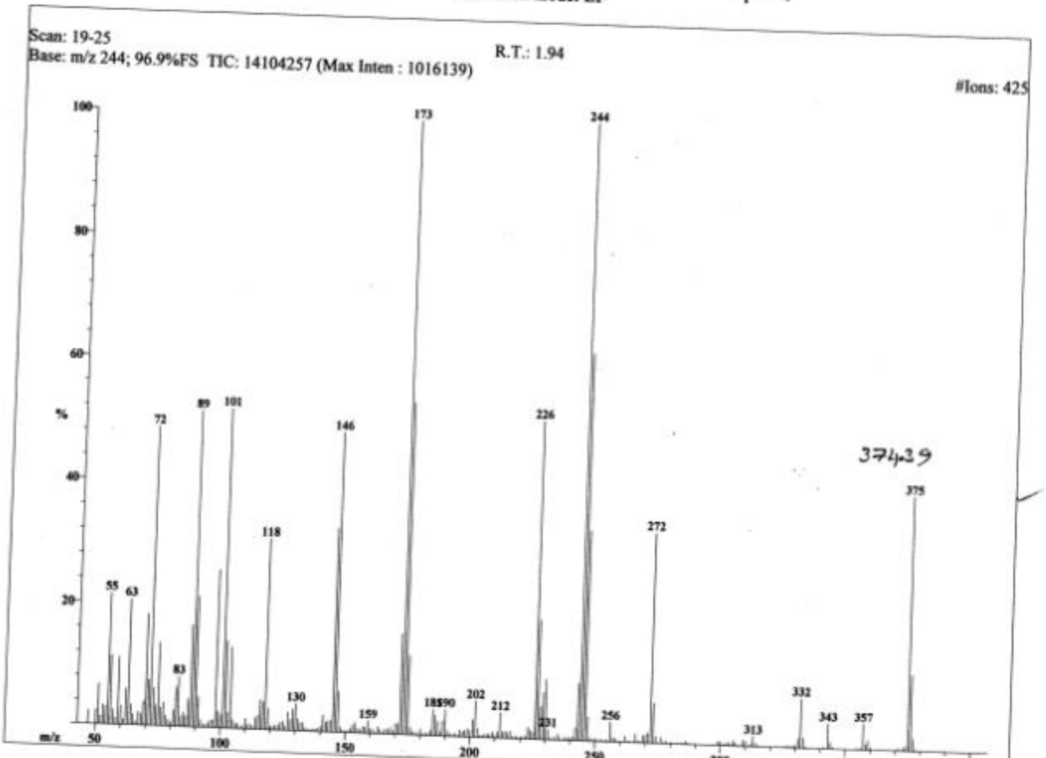

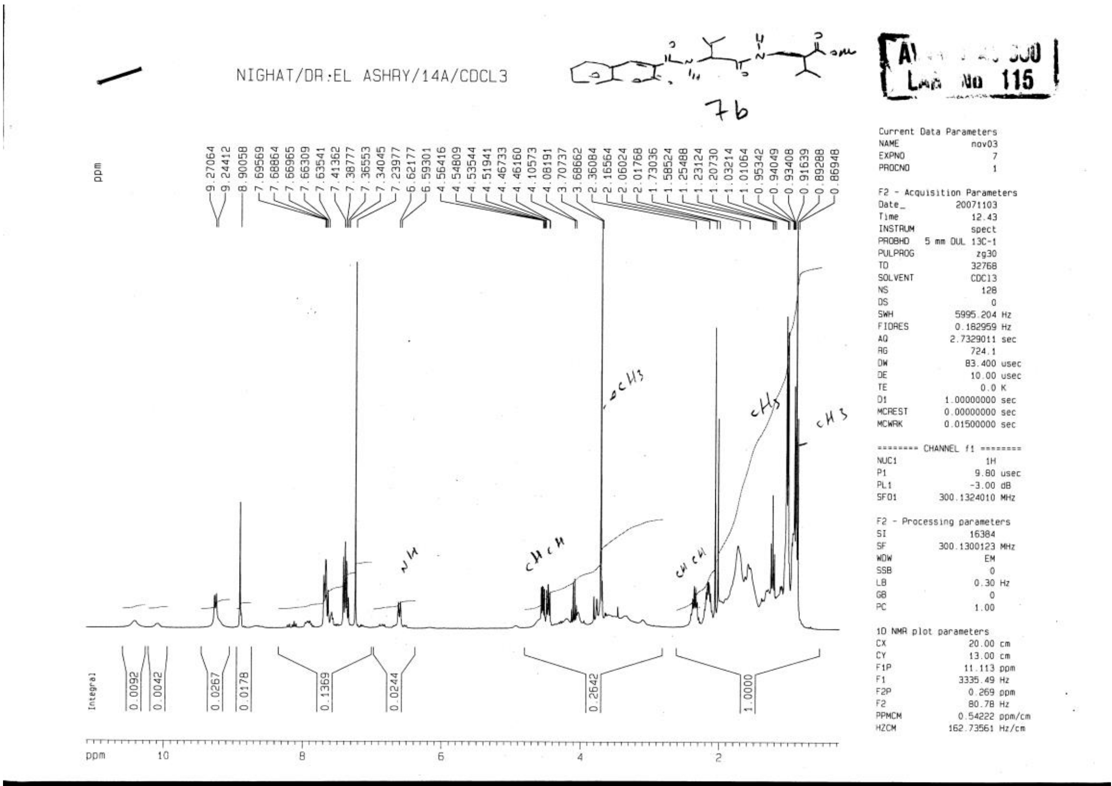

HEJ
11/3/2007

Page 1

File: 14A
Sample: NIGHAT
Instrument: JEOL MSRoute
Inlet: My Inlet

Date Run: 11-03-2007 (Time Run: 14:19:43)

7b

Ionization mode: EI-

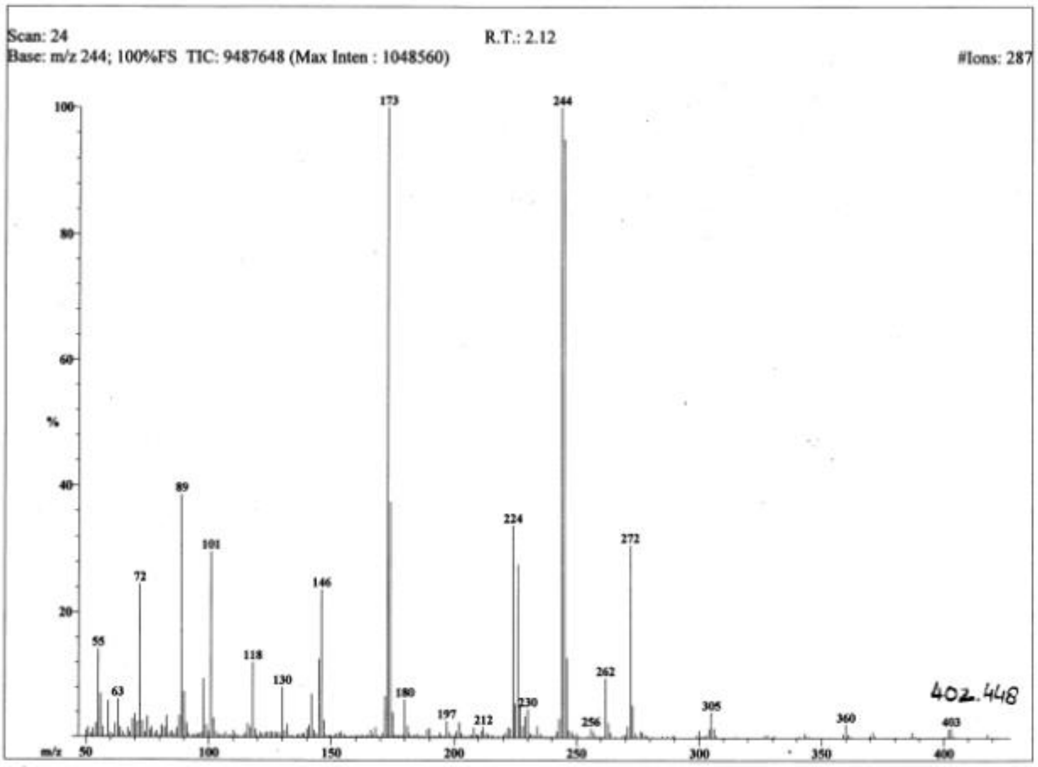

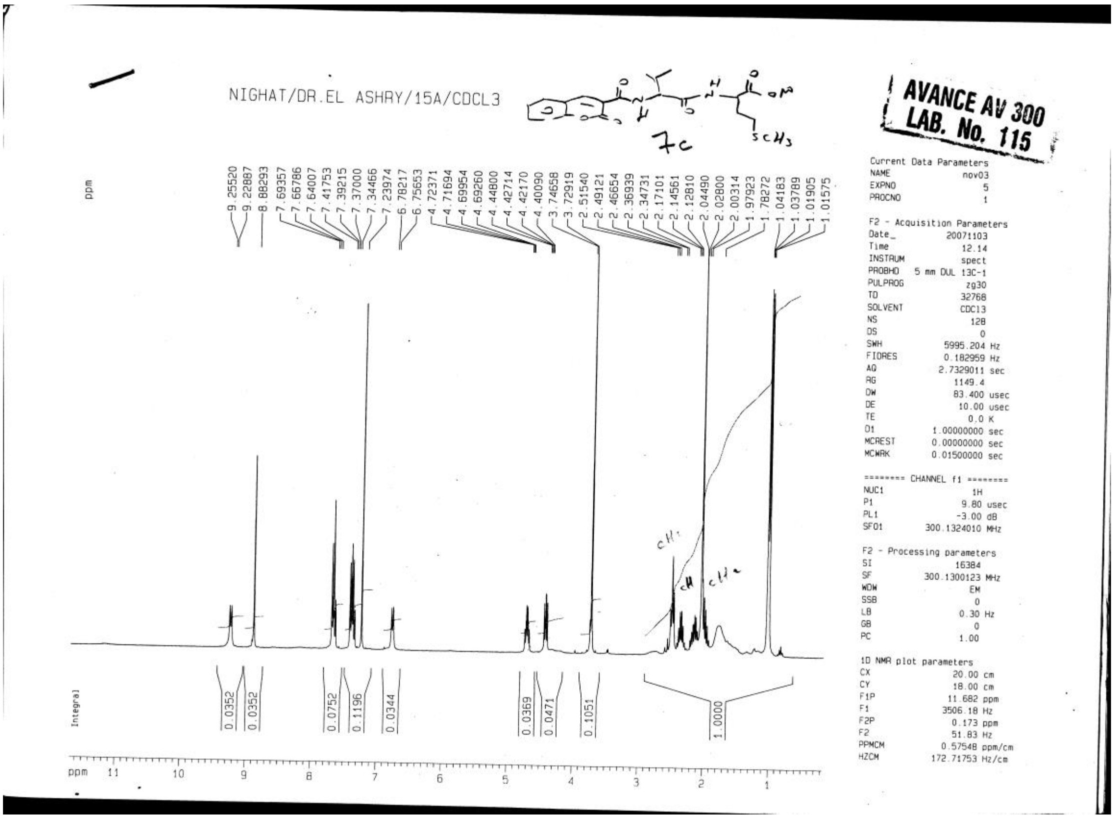

HEJ
11/3/2007

Page 1

File: 15A
Sample: NIGHAT
Instrument: JEOL MSRoute
Inlet: My Inlet

Date Run: 11-03-2007 (Time Run: 14:26:53)

Ionization mode: EI-

7c

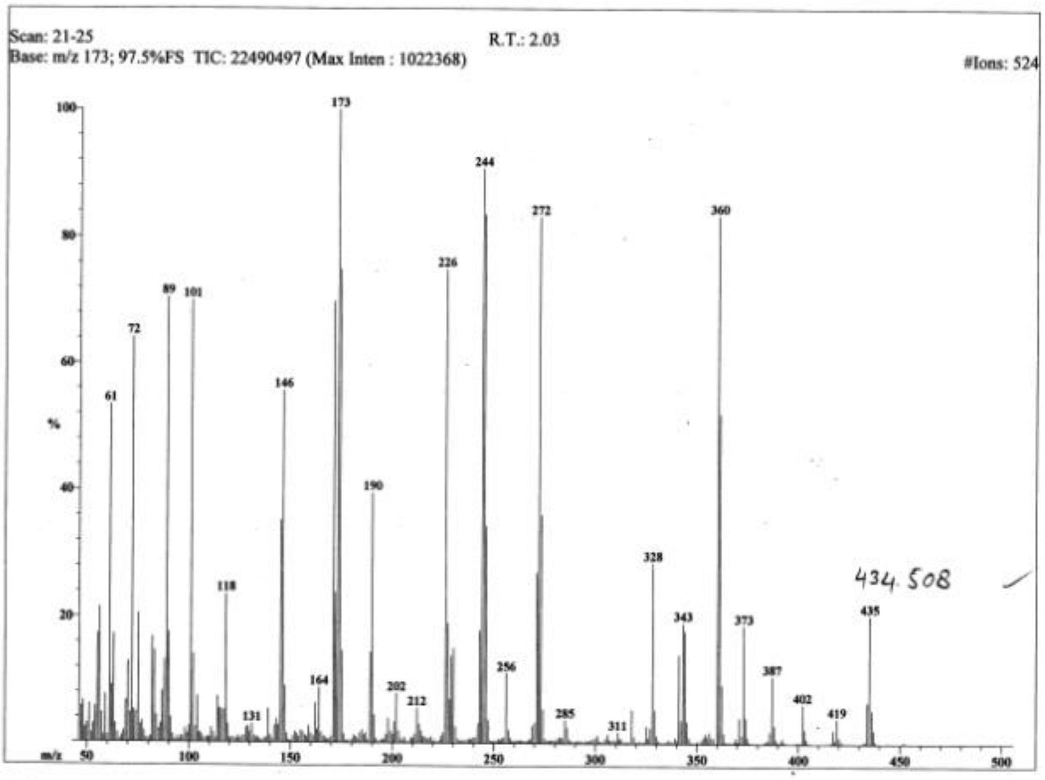

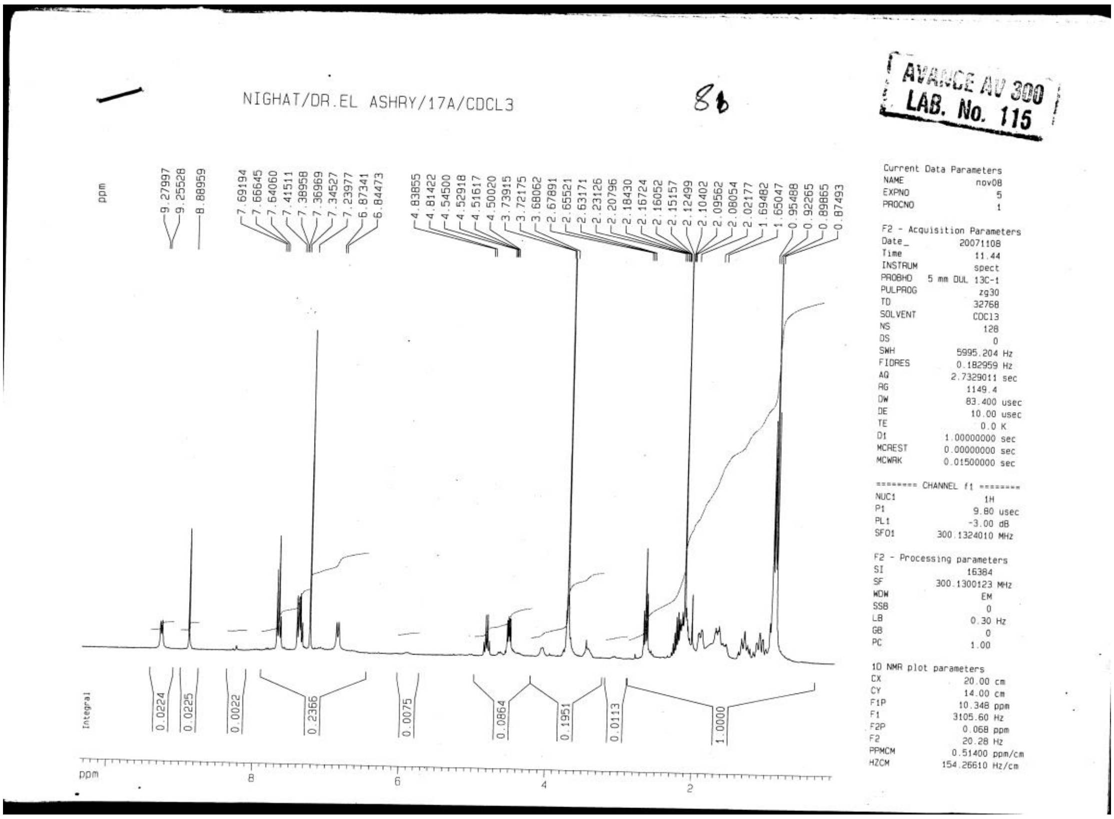
NIGHAT/DR.EL ASHRY/17A/CDCL3
8b
AVANCE AV 300
LAB. No. 115
Current Data Parameters
NAME nov08
EXPNO 5
PROCNO 1
F2 - Acquisition Parameters
Date_ 20071108
Time 11.44
INSTRUM spect
PROBHD 5 mm DUL 13C-1
PULPROG zg30
TD 32768
SOLVENT CDCl3
NS 128
DS 0
SWH 5995.204 Hz
FIDRES 0.182959 Hz
AQ 2.7329011 sec
RG 1149.4
DW 83.400 usec
DE 10.00 usec
TE 0.0 K
D1 1.00000000 sec
MCREST 0.00000000 sec
MCWRK 0.01500000 sec
======== CHANNEL f1 ========
NUC1 1H
P1 9.80 usec
PL1 -3.00 dB
SFO1 300.1324010 MHz
F2 - Processing parameters
SI 16384
SF 300.1300123 MHz
WDW EM
SSB 0
LB 0.30 Hz
GB 0
PC 1.00
1D NMR plot parameters
CX 20.00 cm
CY 14.00 cm
F1P 10.348 ppm
F1 3105.60 Hz
F2P 0.068 ppm
F2 20.28 Hz
PPMCM 0.51400 ppm/cm
HZCM 154.26610 Hz/cm
Integral
0.0224
0.0225
0.0022
0.2366
0.0075
0.0864
0.1951
0.0113
1.0000
ppm
8
6
4
2

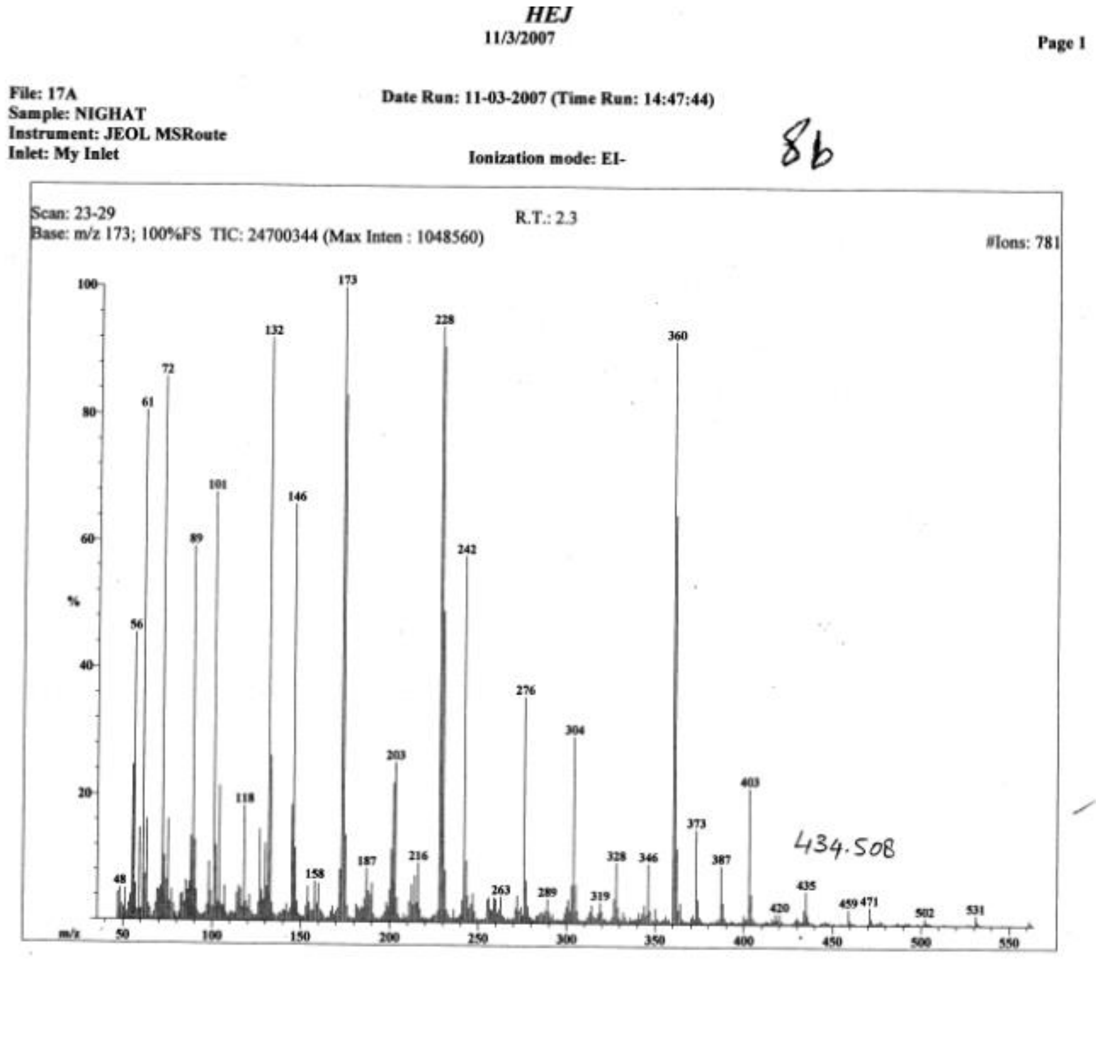
HEJ
11/3/2007
Page 1
File: 17A
Sample: NIGHAT
Instrument: JEOL MSRoute
Inlet: My Inlet
Date Run: 11-03-2007 (Time Run: 14:47:44)
Ionization mode: EI-
8b
Scan: 23-29
Base: m/z 173; 100%FS TIC: 24700344 (Max Inten : 1048560)
R.T.: 2.3
#Ions: 781
%
434.508
m/z
